# Theoretical applications of artificial intelligence in smart infusion pump technology: Expert panel insights

**DOI:** 10.1093/ajhp/zxag085

**Published:** 2026-03-27

**Authors:** Muhammad Mamdani, Kim Walker, Scott D Nelson, Marc Willner, Azizeh Sowan

**Affiliations:** University of Toronto, Toronto, Canada; Yale New Haven Health, New Haven, CT, USA; Department of Biomedical Informatics, Vanderbilt University Medical Center, Nashville, TN, USA; Cleveland Clinic Health System, Cleveland, OH, USA; University of Texas Health at San Antonio School of Nursing, San Antonio, TX, USA

**Keywords:** interoperability, patient safety, smart infusion pumps, workflow efficiency

Infusion pumps are commonly used in hospitals, with more than 70% of hospitalized patients receiving intravenous (IV) infusion treatment.^1^ Smart infusion pumps have significantly advanced patient safety by standardizing infusion practices, reducing medication errors, and providing valuable infusion data that support quality and process improvement.^1-3^ Building on these improvements, interoperability between smart pumps and electronic health records (EHRs) systems has been shown to further reduce medication errors and enhance patient safety.^4^ Although smart pump manufacturers have invested significant effort in enabling integration with EHRs, many health systems have been slow to implement and fully utilize this functionality, with only 9% to 15% of US hospitals implementing pump-EHR interoperability as of 2021.^5-9^ Pump-EHR interoperability is typically enabled through integration with systems such as computerized provider order entry and barcode-assisted medication administration technologies. This integration automates infusion programming and seamlessly documents administered therapies. However, many smart pumps often lack integration with other EHR components, such as laboratory data, medical history, patient vitals, and drug databases, limiting their ability to fully prevent errors.^3^ Further, while smart pumps generate valuable safety data, many healthcare organizations lack the necessary data management and analytics infrastructure to transform this information into meaningful insights that drive continuous improvement.

Artificial intelligence (AI) has the potential to revolutionize healthcare by improving treatment efficiency, diagnostic accuracy, and patient care through advanced data analysis and automation.^10,11^ Leveraging AI’s ability to identify patterns in vast amounts of data could enhance diagnostic precision, optimize clinical workflow, and streamline administrative operations beyond human capacity.^12-14^ However, despite AI’s potential, concerns in areas such as cost, algorithmic bias, model performance, trust, ethics, and integration still limit broader adoption.^15,16^ For example, AI often lacks access to all relevant data for decision-making, which may lead to errors and undermine trust in its reliability.^11,17^ To address some of these challenges, it is crucial to ensure responsible use of AI solutions by keeping humans in the loop to validate recommendations, prevent errors, and maintain trust in clinical decision-making.^10^ Successful implementation of AI-driven solutions requires clear understanding of potential limitations and active engagement from key stakeholders throughout the development and adoption processes.^18^

In smart pump technology, AI could play a crucial role in improving medication administration safety, workflow efficiency, and clinician job satisfaction. To better understand AI potential in smart pump technology, we conducted a panel discussion led by an expert moderator and composed of expert hospital pharmacists, medical informaticists, and nurse informaticists from leading health institutions across the US and Canada. Representing diverse roles and extensive experience, the panel critically examined the clinical utility and feasibility of integrating AI into smart infusion pumps with a focus on patient safety, workflow efficiency, and smart alarms. Through this discussion, the panel identified opportunities for potential AI-based solutions to address current unmet needs, highlighting areas for further innovation and advancement in this space. This paper aims to present the perspectives and insights generated from the expert panel’s discussion.

## Patient safety and medication administration errors

The World Health Organization estimated in 2017 that medication errors injure or harm approximately 1.3 million people in the US annually.^19^ A 2013 review of 91 direct observational studies conducted in hospitals and long-term care facilities found that median medication administration error rates ranged from 8.6% to 28.3%, with notably higher rates (48% to 53%) observed during IV administration, including timing-related errors.^20^ Although current smart pumps incorporate several patient safety features beyond those of a traditional infusion pump—such as dose error reduction software (DERS), predefined drug libraries, and alarm functions—challenges persist, including inaccuracies with drug libraries, IV infusion incompatibility, usability challenges, and the lack of decision support. While some in-house solutions have been developed through tracking of institution-specific metrics and data analysis, many of these concerns remain largely unaddressed with current smart pump technology.^3,21^ Furthermore, even with safety features such as DERS alerting users when preset limits are reached, programming errors still occur.^4^ Smart pumps rely on the user to properly input infusion information into the corresponding pump interface fields and to ensure the infusion is administered to the correct patient.^3^ Many smart pumps have complex user interfaces that require extensive training and challenge users to program them both quickly and accurately, further increasing the risk of pump programming errors. Errors in pump programming are a significant contributor to all medication errors involving the use of smart infusion devices.^3,4^

According to the panel members, medication administration errors may be reduced through EHR integration with smart pumps and AI-driven algorithmic solutions tailored to address these unmet safety needs, with the potential to optimize drug library accuracy, generate actionable insights from pump data, and enable proactive interventions through real-time monitoring and predictive analytics (Table 1). An innovative AI solution could monitor trends in infusion administration data, track pump programming errors and bypassed guardrails/recommended guidelines, reveal patterns behind parameter overrides, pinpoint errors in drug library configurations, and suggest necessary practice adjustments.

**Table 1.** Proposed AI Solutions to Address Patient Safety and Medication Errors

Problem	Proposed AI-driven solution
Frequent pump programming errors	Monitors retrospective administration data (tracking pump programming errors and bypassed guardrails/recommended guidelines) to reveal behavioral patterns/repeated errors in practice that might highlight errors in drug library configurations, potential needs for practice adjustments, and user training
Maintaining an up-to-date drug library	Automated remote updates for new information on medications, compatibility, and guidelines/recommendations
Lack of real-time IV drug compatibility monitoring	Real-time compatibility checks and decision support^a^ Secondary solution: Supporting evidence generated for infusion combinations and drug compatibilities that are currently lacking in literature^a^
Overly broad drug library parameters	Patient profiling: group/select (preprogrammed) types of patients with common diagnoses/characteristics that often need a different range of a drug than the guideline or recommendation based on patient health conditions and treatment care area
Lack of clinical decision support	Dynamic, real-time treatment recommendations by monitoring patient through infusion and all available inputs (eg, diagnoses, vitals, lab results, raw medical images, etc)^a^ Secondary solution: AI-driven predictive analytics to identify patients at risk for adverse events, prolonged hospital stays, mortality, etc^a^

Abbreviations: AI, artificial intelligence; EHR, electronic health record; IV, intravenous.

^a^Requires interoperability with EHR.

Maintaining an up-to-date drug library requires continuous monitoring and regular updates, especially with changing treatment protocols across care areas. Errors can occur as a result of outdated guidelines, missing compatibility information, or a lack of standardization across hospital networks and care areas. There may be benefits to implementing a solution that monitors emerging literature and recommends drug library updates based on newly issued guidelines and recommendations. With IV compatibility playing a crucial role in medication errors with smart pumps, integrating real-time compatibility checks and decision support could further optimize patient safety and care. Additionally, for medication combinations that lack published infusion compatibility (or incompatibility) data, AI could be leveraged to track each infusion trend, document administration combinations and outcomes, and build “new” evidence to inform future compatibility recommendations (Table 1).

Drug library parameters are often defined broadly to minimize alarms and increase workflow efficiency for the widest possible range of infusions and patient populations. However, broad definitions, while accommodating for outliers, reduce safety for patients by creating space for overtreatment within the allowable parameters of the drug library. AI could be enabled to identify and segment patient populations with shared diagnoses/characteristics who frequently require drug dosing outside the standard guideline range, or have unique needs, while maintaining narrowed standard parameters for the majority. In such a solution, adjusted guidelines/recommendations within the drug library would be restricted to only a particular group of patients rather than applied to all patients. For example, opioids, insulin, and heparin may be particularly suited to this model as these medications are programmed to be monitored under broad ranges, of which extremes may not be completely appropriate for some patients and present heightened potential for severe harm but are needed in a select subset of patients. Such a solution could allow safer ranges for both the general patient population and those with specialized needs.

Current smart pumps are largely reactive, rather than predictive, and lack advanced clinical decision support beyond basic dose-checking alerts. Integrating real-time monitoring with multimodal AI that processes a wide range of the collected patient data, such as diagnoses, vitals, laboratory results, and raw medical images, could facilitate dynamic, real-time treatment recommendations and predictive care. AI-driven predictive analytics within smart pumps could identify infusion-related risks and potential adverse events, optimize IV medication administration, and trigger timely clinical decisions to improve patient safety. This, in turn, may lead to faster recovery and efficient utilization of healthcare resources.

## Workflow efficiency and smart alarms

Hospital workflow efficiency is significantly constrained by clinician time, available resources, and device functionality. Panelists unanimously agreed that the top priority should be to develop a strategy ensuring only clinically meaningful alarms are triggered (Table 2). Bedside clinicians experience a large quantity of clinically insignificant alarms and alerts, leading to desensitization and frequent alarm silencing and dismissals.^22^ One study found users in the intensive care unit (ICU) setting dealt with an average of 45.5 alarms per patient per hour, with infusion pumps contributing to 9.6% of the total alarm burden.^23^ Further, infusion pump alarms had a positive predictive value lower than that of all medical devices combined (7.6% vs 36.2%).^23^ Alarm fatigue occurs as clinicians are continually exposed to overwhelming, frequent, and inactionable alerts, impacting clinicians’ already limited time. A retrospective study evaluating alarm alerts in smart infusion data found that infusions averaged 2 operational alarms per individual infusion and 1 programming alert for every 48 infusions.^24^ Additionally, while the mean resolution time for 74.5% of alarms was 1 minute or less, 8% of alarms took more than 4 minutes to be resolved.^24^ Dedicating time to investigate false alarms demands unnecessary interventions, diverts time and attention from critical tasks, and increases the cognitive burden placed on clinicians, which may lead to delayed responses in other areas of care and increased risk of human errors. The extensive number of alarm alerts and alarm dismissals not only impacts bedside clinicians but also heightens patient dissatisfaction, anxiety, and reduced sleep quality.^25,26^ Pharmacists and decision-makers may also feel the effects downstream as they try to analyze scenarios to uncover issues, such as dispensing order failures resulting from data discrepancies, retrospectively. AI could be leveraged to automate checks for data alignment across all interoperable inputs, cross-reference the information with pharmacy dispensing orders, and avoid delays resulting from documentation errors. This would further streamline workflow efficiency and reduce the strain on clinicians, potentially improving overall job satisfaction. Further, AI could assimilate national benchmark data, such as alarm rate analytics conducted by the National Infusion Collaborative (Bainbridge Health, Philadelphia, PA), to understand error patterns and identify additional opportunities for the safe reduction of smart pump alarms.

**Table 2.** Proposed AI-Driven Solutions to Address Workflow Efficiency and Create Smart Alarms

Problem	Proposed AI-driven solution
Understanding clinician response to alarms	Tracks maneuvers taken after a sound alarm (identifying patterns in behaviors such as frequently dismissed alerts) to evaluate and define appropriate responses to alarms Secondary solution: Provides real-time guidance for appropriate responses to alarm alerts and troubleshooting
Frequent inactionable or false alarms	Adjusts or maintains infusions dynamically, within appropriate parameters, based on active patient parameters^a^
Excessive documentation time and risk of errors	Auto-documents pump administration data, and reconciles with EHR documentation and dispensing order^a^
Clinicians’ limited time	Real-time monitoring and prioritizing^a^ of alarms through remote mobile device, enabling instant access to infusion data and continuous patient oversight without requiring frequent bedside checks Secondary solution: Track pump metrics, pump device allocation, and future dispensing needs through device analytics to illustrate supplemental areas for improving efficiency
Anticipated timely smart pump device maintenance	Monitors infusion pump performance and device health through predictive remote maintenance

Abbreviations: AI, artificial intelligence; EHR, electronic health record.

^a^Requires interoperability with EHR.

While it is important to track the alarm frequency and response time, decision-makers are most interested in understanding the reason for alarm dismissal and the actions taken directly after an alarm has sounded. A solution to address this might be 2-fold: first, creating an AI-driven solution that tracks these “next step” maneuvers after an alarm to understand the actions being taken, and second, leveraging that information to design an AI system that provides direction and guidance for appropriate responses to alarm alerts and real-time troubleshooting. Implementation of AI-powered algorithms that dynamically adjust infusions within appropriate parameters that are based on active patient metrics may combat time limitations through proactive alarm resolution, but smart pumps would need to be interoperable with input metrics to aggregate all necessary patient information for safe and effective decision-making.

With the technological push for interoperability between pump and EHR, solutions that facilitate auto-documentation may provide further time savings. A real-time monitoring solution accessible through a remote mobile device could also help mitigate clinician time constraints by providing instant access to infusion data, enabling continuous patient oversight without frequent bedside visits, and offering recommendations to prioritize clinical actions. Analytics gathered through these devices could track pump metrics and pump device allocation and be used to anticipate future dispensing needs to illustrate supplemental areas for improving efficiency and workflow. Furthermore, AI-powered predictive maintenance could monitor smart pump performance and device health, detecting potential failures early to ensure that all pumps remain safe and up-to-date for clinical use.

## Priority of solutions

When developing and implementing AI solutions, it is often essential to keep humans in the loop, especially in a setting of care. AI should be positioned to enhance and supplement human decision-making rather than replace it, as current AI systems, while able to outperform clinicians in certain tasks, have not yet demonstrated the ability to consistently improve clinical reasoning in complex scenarios as standalone aids.^27^ This importantly highlights the need for thoughtful integration and continued development of both technology and clinical workflows. During development, a primary focus should be on understanding the specific problems within current workflows that solutions are aiming to address, rather than rushing to evaluate or deploy solutions. By first addressing high-impact, low-effort initiatives, healthcare facilities can improve care quality and efficiency while establishing a strong foundation for future advancements. Once these foundational improvements are in place, more advanced and automated AI solutions may be introduced. This phased strategy will allow healthcare organizations to adapt to emerging technologies while maintaining operational stability.

## Timeline and expectations of AI

The complexity of AI solutions impacts the estimated timeline for developing and deploying new solutions, as it requires extensive time for algorithm design, testing, validation, and regulatory approval before clinical implementation. There are several attainable solutions that can be implemented with relatively minimal efforts to improve efficiency and patient safety. These include tracking trends in medication administration data, maintaining up-to-date drug libraries, utilizing benchmarking data, and alerting clinicians about potential IV incompatibilities. In general, patient safety initiatives may be prioritized over efficiency-focused solutions, as safety remains a foundational aspect of healthcare with a direct impact on patient outcomes, whereas efficiency, while important, is more closely tied to operational performance. Many healthcare facilities are already collecting vast amounts of data, and with relatively small adjustments, these data could be packaged into useful solutions that support clinical decision-making. While AI-powered solutions like dynamic dose adjustment and real-time remote monitoring could enhance infusion pump performance, these solutions require interoperable inputs. Integrating these data could significantly improve patient care by providing clinicians with real-time insights and alerts.

## Considerations and barriers to AI adoption

To our knowledge, there are no existing AI algorithms that have been implemented into smart pump technologies. Though, a few retrospective studies have investigated the potential for machine learning to improve infusion pump device safety and reliability.^28,29^ One case study tested and established the feasibility of several machine learning methods in predicting device performance and potential failures through retrospective pump inspection data—all methods showcased satisfactory results in all validation performance metrics.^28^ The study’s conclusions stressed the need for transforming the current reactive approach to infusion pump management into a proactive (real-time) and predictive one.^28^ Considerable exploration into the feasibility and development of AI within other medical technologies, such as a deep learning algorithm for accurately predicting anesthesia depth or the creation of fully automated “closed-loop” insulin delivery systems for diabetes management, has generated interesting and relatively translatable insights that may help blueprint approaches to AI and machine learning innovation in infusion pumps.^30,31^ Advances in reinforcement learning, which allow systems to learn through experience/feedback in their environment, have enabled AI-enhanced technologies within insulin management to adapt to individual variability and dynamic factors such as insulin absorption and glucose response.^30^ While promising, further research into the complex interactions of all variables involved in insulin response, and medication delivery as a whole, remains an important step as these AI advancements and methods evolve.

Adopting AI solutions in smart pump technology faces several key barriers. One major challenge is cost, as institutions need verified return on investment (ROI) demonstrated through positive clinical and health economic outcomes. A report from the Betsy Lehman Center in 2019 estimated the average cost of an adverse drug event to be $3,260.^32^ Given the significant upfront expenses—ranging from device acquisition to technological integration and staff training—institutions must justify these investments with clear, quantifiable benefits. Without robust data proving cost savings or revenue generation, stakeholders may be reluctant to prioritize smart pump initiatives over competing capital needs. Therefore, concrete, real-world evidence demonstrating reduced errors, operational efficiencies, and financial gains is essential to drive broader adoption.

Concerns regarding trust in ethical AI use are another significant hurdle. Patient privacy, data security, transparency of algorithmic decisions, and bias must all be carefully considered, both institutionally and governmentally, during the development and implementation of AI in healthcare. Clinicians must feel confident in the accuracy of AI outputs, which requires thorough quality checks such as algorithmovigilance for drift detection and clinician verification (approval/rejection) of recommendations. Algorithmic bias, stemming from existing bias in training data through the historical absence or misrepresentation of certain populations, may present itself differently across health specialties and may exacerbate existing inequities. Developers and clinicians share a duty to ensure that training sets are representative, model outputs are audited for fairness, and potential inequities and biases are monitored continuously post deployment.

The effort involved in adopting and integrating AI solutions can be substantial, often requiring phased implementation with clear timelines/milestones as well as additional considerations for special populations (eg, pediatrics). Phased exploration and implementation may be warranted for the adaptation of solutions to new specialties and populations and should require continuous input from all relevant stakeholders. Continuous quality improvement initiatives examining best practices are essential for upholding cutting-edge innovation and ensuring the effectiveness of processes. Hesitation or resistance from key stakeholders—including nurses, pharmacists, physicians, biomedical engineers, quality and safety leaders, informatics teams, and the healthcare system leadership—is natural and expected as new technologies are introduced.^12,33-35^ These groups share concerns related to patient safety, workflow efficiency, and system reliability. As with all new technological advancements, any proposed AI solutions should be designed, and eventually implemented and operated, in collaboration with end users rather than solely on their behalf.

Understanding current clinician performance is critical when implementing new technology. For example, a study performed at Unity Health Toronto assessing physicians’ ability to predict in-hospital death or the need for intensive care found that physicians were correct less than a third of the time; this led to the development of ChartWATCH, a machine learning algorithm that predicts patient ICU admissions.^36^ Identifying a poor level of prognostic performance not only made clinicians more receptive to leveraging a supportive AI tool, but also objectively set a standard for AI performance in this setting—to perform at or better than this established level. Patient variability in critical care complicates mortality prediction, and the ChartWATCH algorithm offers an additional resource to physicians for their comprehensive assessment and to pinpoint information gaps. Among interactions with infusion pumps, performance metrics may be demonstrated through alarm analytics and cross-referenced alongside benchmark metrics, whether through health-system standards or through data collaboration alliances such as the National Infusion Collaborative. Benchmark metrics are valuable for illustrating the full picture of institutional success, establishing a baseline for comparison, and understanding real-world adoption and employment of solutions and technologies.

## Conclusion

While significant improvements have been made in infusion-related patient safety, there are still unmet needs that present opportunities for AI, including drug library optimization, real-time monitoring, and predictive care. Implementing AI has the potential to enhance efficiency and reduce alarm fatigue, ultimately improving clinician job satisfaction through solutions like auto-documentation, dynamic dose adjustments, alarm analyses, and decision support systems. Although these fully automated, closed-loop AI solutions remain a future goal, more immediate benefits can be achieved by consolidating and organizing existing data. Much of the data needed to support these solutions is already being collected by smart infusion pumps, providing a foundation for AI to organize and analyze data and generate valuable insights. While many AI solutions have great potential, addressing smaller, more attainable opportunities first may be the best approach before tackling larger, more complex projects. A key hurdle to AI adoption is the upfront cost and uncertainty about ROI, and while interoperability is crucial for more advanced analytics, AI can still provide value through solutions like drug library optimization even without full interoperability.

## Data Availability

All data underlying this article are available in the article.
